# Progression of radiographic fibrosis in rheumatoid arthritis-associated interstitial lung disease

**DOI:** 10.3389/fmed.2023.1265355

**Published:** 2023-09-22

**Authors:** Dandan Chai, Di Sun, Yuanying Wang, Yawen Song, Na Wu, Qiao Ye

**Affiliations:** ^1^Clinical Center for Interstitial Lung Diseases, Beijing Institute of Respiratory Medicine, Beijing Chaoyang Hospital, Capital Medical University, Beijing, China; ^2^Department of Occupational Medicine and Toxicology, Beijing Chaoyang Hospital, Capital Medical University, Beijing, China

**Keywords:** rheumatoid arthritis, interstitial iung preclinical, preclinical interstitial iung disease, pulmonary fibrosis, risk factor

## Abstract

**Background and objectives:**

Preclinical interstitial lung disease (pILD) may represent the early stages of rheumatoid arthritis-associated interstitial lung disease (RA-ILD). However, the characteristics, clinical outcomes, and risk factors associated with fibrosis progression in RA-ILD, including pILD and ILD, remain poorly understood.

**Methods:**

Baseline data were compared between patients with RA-ILD and those with RA alone. Multivariate logistic regression and Cox regression analyses were performed to identify risk factors associated with the prevalence and imaging progression of RA-ILD, respectively.

**Results:**

Among the 371 enrolled RA patients, 32.3% had RA-ILD. Multiple logistic regression analyses identified age over 60.0 years (OR 2.22), smoking (OR 2.09), diabetes mellitus (DM) (OR 3.09), mixed connective tissue disease (MCTD) (OR 2.98), serum lactate dehydrogenase (LDH) levels exceeding 250.0 U/L (OR 6.73), and positive anti-cyclic citrullinated peptide (anti-CCP) antibody (OR 2.06) as independent risk factors for RA-ILD (*p*< 0.05 or 0.01). Among the 98 RA-ILD patients who underwent follow-up for a median duration of 19.1 months, 51.0% demonstrated fibrotic progression on high-resolution computed tomography (HRCT). Multiple Cox regression analysis identified DM (HR 2.03), Disease Activity Score in 28 joints-Erythrocyte Sedimentation Rate (DAS28-ESR) greater than 5.1 (HR 2.21), and baseline HRCT scores exceeding 5.0 (HR 2.30) as independent risk factors for fibrosis progression in RA-ILD (*p*< 0.05 or 0.01).

**Conclusion:**

Nearly one-third of RA patients in this cohort had prevalent pILD or ILD, and half of them demonstrated imaging progression during follow-up. DM, higher DAS28-ESR, and advanced HRCT scores were identified as independent risk factors for progressive fibrosis in RA-ILD.

## Introduction

Rheumatoid arthritis (RA) is a systemic autoimmune disease that affects approximately 0.5 to 1.0% of the general population. The exact pathogenesis of RA is not fully understood, but it is believed to involve autoimmune and inflammatory responses, as well as environmental factors that contribute to the prevalence and progression of the disease (1). The initial manifestation of RA is typically characterized by symmetric polyarticular pain and swelling. Additionally, patients often experience nonspecific systemic symptoms such as fatigue, low-grade fever, muscle aches and pains, or weight loss (2). However, RA is not limited to joint involvement alone; it is a systemic inflammatory disease that can affect various organs, including the heart, lungs, skin, and eyes. The presence of extra-articular involvement in approximately 17.8–40.9% of RA patients can significantly impact their prognosis (2, 3).

Rheumatoid arthritis has the potential to substantially impact the lung parenchyma, presenting as interstitial lung disease (ILD), as an extra-articular manifestation that exhibits a heterogeneous condition characterized by diverse pathological manifestations and ultimately culminating in irreversible fibrosis; this association is linked to notable morbidity and mortality (4, 5). The reported prevalence of RA-ILD varies widely, ranging from 1.0 to 58.0%, reflecting differences in study designs and inclusion criteria (6). Advanced age, male gender, tobacco exposure, and the presence of positive rheumatoid factors (RF) or anti-cyclic citrullinated peptide (CCP) antibodies have been identified as risk factors for the development of RA-ILD (7). Respiratory symptoms commonly observed in RA-ILD resemble those seen in idiopathic interstitial pneumonia (IIP) and include dyspnea, cough, chest tightness, shortness of breath, and velcro crackles at the base of both lungs (8). In advanced stages, pleural friction sounds and pulmonary hypertension may also be present (8). Although overall mortality rates in RA have decreased, ILD has emerged as a leading cause of death in RA patients, second only to cardiovascular complications (9).

Preclinical ILD (pILD) refers to the incidental discovery of interstitial lung abnormalities on chest high-resolution computed tomography (HRCT) scans in individuals without a prior diagnosis of ILD (10). pILD exhibits resemblances to ILD, potentially signifying its early stages, such as sharing radiological findings characteristic of early ILDs (11–13). Patients with interstitial lung abnormalities can be divided into two categories: incidental interstitial lung abnormalities (ILA), which are detected during chest HRCT scans conducted for purposes such as cancer screening or general health examinations, and pILD, which is identified during screening for ILD in high-risk groups (such as those with connective tissue diseases or familial ILD) (14). Based on distinct imaging features and distributions, pILD can be classified into three patterns: non-subpleural non-fibrotic, subpleural non-fibrotic, and subpleural fibrotic, with an increasing risk of progression and mortality in that order (15). Fibrosis is characterized by lung distortion with traction bronchiectasis and/or honeycombing, consistent with the fibrosis criteria outlined in the latest guidelines for idiopathic pulmonary fibrosis (IPF) and progressive pulmonary fibrosis (PPF) in 2022 (16).

However, the characteristics and clinical outcomes of RA-ILD, including pILD and ILD, remain poorly understood. The objective of this study was to investigate the prevalence, clinical features, prognosis, and risk factors associated with the imaging progression of RA-ILD, encompassing both pILD and ILD.

## Methods

### Study design and participants

This retrospective cohort study was conducted at Beijing Chao-Yang Hospital, a regional tertiary referral center specialized in the clinical and research of ILDs. The patients aged ≥18 years hospitalized patients with RA from January 1, 2017, to December 31, 2021 were screened consecutively. The diagnosis of RA was made by rheumatologists in accordance with the 2010 American College of Rheumatology/European League Against Rheumatism criteria for RA classification (17). Given the constraints of the local medical insurance policy and the personal circumstances of the patients, the hospitalized patients offer a more comprehensive set of clinical data for evaluating joint conditions and extra-articular organ involvement. Patients who had undergone clinically indicated and interpretable chest HRCT scans were included in this study. Multidisciplinary diagnoses were conducted between pulmonologists, radiologists, rheumatologists, and pathologists experienced in the diagnosis of ILD based on clinical characteristics, HRCT, and lung biopsy if appropriate. The following exclusion criteria were applied: (1) uncontrolled lung infection, (2) decompensated heart failure, (3) current or prior malignancy, and (4) absence of chest HRCT.

**Figure 1:**
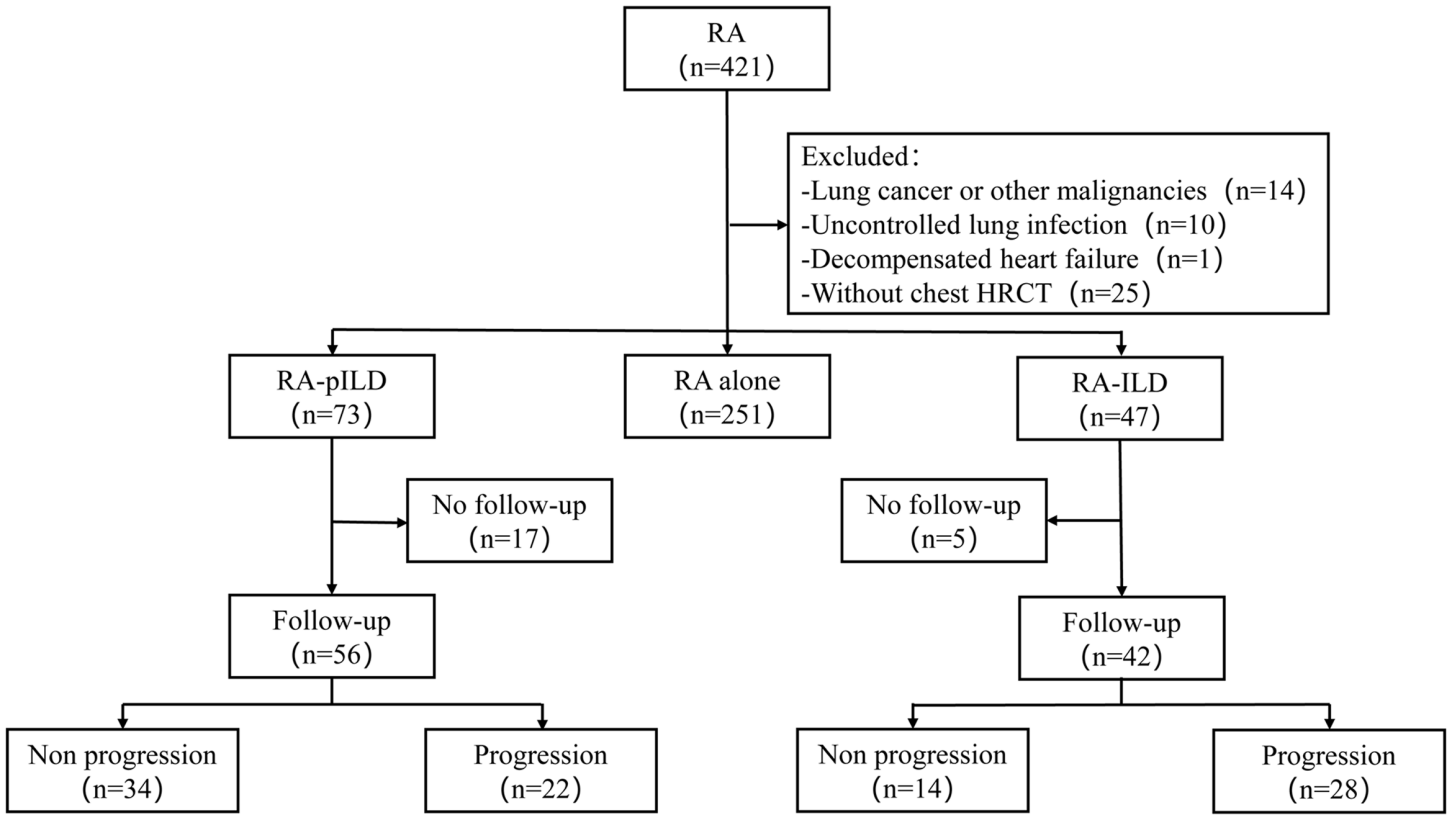
Flowchart of the study. RA, rheumatoid arthritis; HRCT, high-resolution computed tomography; ILD, interstitial lung disease; pILD, preclinical interstitial lung disease.

Ethical approval for this study was obtained from the Ethics Committee of Beijing Chao-Yang Hospital (2023-scientific-644). A detailed flow diagram illustrating the study’s methodology is presented in Figure 1. All procedures were conducted in accordance with the principles outlined in the Declaration of Helsinki.

### Data collection

All patient information was extracted from medical records, encompassing demographic details, HRCT imaging results, pulmonary function tests, laboratory data, and therapy regimens. Disease activity was evaluated using the Disease Activity Score in 28 joints-Erythrocyte Sedimentation Rate (DAS28-ESR). Inflammation markers derived from blood cell counts included the neutrophil-to-lymphocyte ratio (NLR), monocyte-to-lymphocyte ratio (MLR), platelet-to-lymphocyte ratio (PLR), systemic inflammatory index (SII = neutrophils × platelets/lymphocytes), systemic inflammatory response index (SIRI = neutrophils × monocytes/lymphocytes), and aggregate index of systemic inflammation (AISI = neutrophils × platelets × monocytes/lymphocytes). Seronegative RA refers to RA patients who exhibit clinical manifestations associated with RA, such as morning stiffness, joint swelling and pain, elevated C-reactive protein (CRP) and erythrocyte sedimentation rate (ESR), despite negative anti-CCP antibody and RF results.

### Imaging description and follow-up

Two experienced pulmonologists, blinded to the clinical data, independently reviewed the chest HRCT scans. They categorized individuals into two groups: RA alone and RA-ILD (including pILD and ILD), and visually scored the HRCT scans. Any disagreements between the observers were resolved through consultation. Each lung was divided into three zones, and a score ranging from 0 to 24 points was assigned based on the percentage of abnormal imaging findings in each lung zone. The interobserver agreement, measured using weighted kappa, was found to be 0.83.

Patients diagnosed with RA-ILD (including pILD and ILD) were followed up in the clinics and reexamined the chest HRCT every 6–12 months for monitoring the abnormalities of ILD. The follow-up period ended on December 31 2021. Based on the imaging findings, the patients were categorized as either progressors or non-progressors. Progression was defined as the appearance of new imaging features and/or an increase in the extent or density of abnormal imaging compared to the initial scan (18, 19). The outcome of the study was the imaging progression of RA-ILD. Patients who underwent a follow-up chest HRCT after the study’s end date were not included in the analysis. Additional methodological details can be found in the Supplementary Appendix.

### Statistical analysis

Data analysis was conducted using SPSS 26.0 statistics software. Measurement data were presented as mean (standard deviation, SD) for variables with a normal distribution, while median (interquartile range, IQR) was used for variables without a normal distribution. The Mann–Whitney *U* test or *t*-test was employed to compare continuous variables between groups, while the chi-squared test or Fisher’s exact test was used for categorical variables. Multivariate logistic regression and Cox regression analyses were performed to identify risk factors associated with the prevalence and imaging progression of RA-ILD, respectively. A two-sided *p*-value <0.05 was considered statistically significant, indicating a significant difference.

## Results

### Demographics and baseline characteristics of the patients

Among the 371 RA patients who met the inclusion criteria, 251 (67.7%) had RA alone, while 120 (32.3%) had RA-ILD (including pILD and ILD). The median age of the entire cohort was 62.0 (IQR 54.0–71.0) years, with 30.7% being males and 27.0% being current or ex-smokers. Patients with RA-ILD exhibited a higher proportion of individuals aged >60.0 years, males, smokers, and a higher prevalence of comorbid diabetes mellitus (DM) or mixed connective tissue disease (MCTD). They also presented respiratory symptoms and signs, all of which showed statistically significant differences (all *p*< 0.05). In terms of the proportion of values above the upper limit of normal, the RA-ILD group had higher levels of DAS28-ESR (defined as DAS28-ESR > 5.1) (*p*= 0.04), serum ferritin (*p*= 0.01), lactic dehydrogenase (LDH) (*p*< 0.01), and positive anti-CCP antibody (*p*= 0.04) compared to the RA alone group. Additionally, the RA-ILD group had significantly lower values of forced vital capacity (FVC)% predicted, diffusion capacity of the lungs for carbon monoxide (DLCO)% predicted, and partial pressure of arterial oxygen (PaO2) (all *p*< 0.01) (see Table 1). Among 120 RA-ILD (including pILD and ILD) patients, the patients with RA-ILD showed more symptoms and signs, higher HRCT scores and poorer pulmonary function (*p*< 0.01 or 0.05) (see Supplementary Table S1).

**Table 1:** Demographics and baseline characteristics of RA patients.

Variables	All	RA-ILD^a^	RA alone	*p*-value
*n*	371	120	251	
Age, years	62.0(54.0–71.0)	67.0(60.0–75.0)	60.0(52.0–67.0)	**<0.01**
Male, *n* (%)	114(30.7)	46(38.3)	68(27.1)	**0.03**
BMI, kg/m^2^	24.0 ± 3.8	24.1 ± 3.6	23.9 ± 3.9	0.62
Smoking status				**<0.01**
Smoker, *n* (%)	100(27.0)	44(36.7)	56(22.3)	
Non-smoker, *n* (%)	271(73.0)	76(63.3)	195(77.7)	
Pack years	23.0(15.0–40.0)	30.0(15.0–43.8)	20.0(15.0–40.0)	0.33
Symptoms and signs, *n* (%)				
Dyspnea	49(13.2)	45(37.5)	4(1.6)	**<0.01**
Cough	73(19.7)	55(45.8)	18(7.2)	**<0.01**
Velcro crackles	46(12.4)	45(37.5)	2(0.8)	**<0.01**
Laboratory findings				
DAS28-ESR	4.7 ± 1.8	4.9 ± 2.1	4.6 ± 1.6	0.24
DAS28-ESR, *n* (%)				**0.04**
≤5.1	220(59.3)	62(51.7)	158(62.9)	
>5.1	151(40.7)	58(48.3)	93(37.1)	
ESR, mm/h	27.0(15.0–45.0)	26.0(15.3–44.0)	28.0(15.0–45.0)	0.79
ESR, *n* (%)				0.92
≤15 mm/h	94(25.3)	30(25.0)	64(25.5)	
>15 mm/h	277(74.7)	90(75.0)	187(74.5)	
CRP, mg/dl	1.2(0.5–3.7)	1.1(0.5–3.5)	1.2(0.5–3.8)	0.80
CRP, *n* (%)				0.90
≤0.8 mg/dl	147(39.6)	47(39.2)	100(39.8)	
>0.8 mg/dl	224(60.4)	73(60.8)	151(60.2)	
Ferritin, ng/ml	160.5(77.0–254.2)	178.6(110.5–318.2)	147.9(66.0–240.1)	**<0.01**
Ferritin, *n* (%)				**<0.01**
≤322 ng/ml	309(83.3)	91(75.8)	218(86.9)	
>322 ng/ml	62(16.7)	29(24.2)	33(13.1)	
LDH, U/L	185.0(161.0–222.0)	214.5(178.3–267.0)	176.0(155.0–206.0)	**<0.01**
LDH, *n* (%)				**<0.01**
≤250 U/L	319(86.0)	82(68.3)	237(94.4)	
>250 U/L	52(14.0)	38(31.7)	14(5.6)	
RF positivity, n (%)	263(70.9)	88(73.3)	175(69.7)	0.47
RF titer, IU/ml	159.0(55.1–482.0)	174.0(55.5–405.8)	135.0(51.8–535.0)	0.70
Anti-CCP positivity, *n* (%)	278(75.7)	99(82.5)	179(72.5)	**0.04**
Anti-CCP titer, IU/ml	768.8(212.9–2047.7)	749.3(274.6–2050.6)	788.0(203.8–2046.8)	0.57
Seronegative RA^b^, *n* (%)	60(16.2)	11(9.2)	49(19.5)	**0.01**
NLR	2.8(1.9–4.0)	3.1(2.1–4.1)	2.6(1.8–3.9)	**0.02**
MLR	0.3(0.2–0.4)	0.3(0.2–0.4)	0.3(0.2–0.4)	0.22
PLR	160.4(111.9–231.8)	141.4(99.0–190.5)	169.9(119.3–249.5)	**<0.01**
SII	711.2(422.2–1120.9)	753.9(422.2–990.6)	702.8(420.2–1176.8)	0.89
SIRI	1.1(0.7–1.9)	1.4(0.8–2.4)	1.0(0.7–1.8)	**<0.01**
AISI	310.7(143.7–536.9)	346.7(148.4–635.2)	296.3(141.6–534.8)	0.22
Pulmonary function				
FVC, %predicted	100.3 ± 22.0	96.3 ± 20.7	104.8 ± 22.6	**<0.01**
DLCO, %predicted	73.5 ± 18.0	65.8 ± 15.6	82.7 ± 16.4	**<0.01**
PaO2, mmHg (room air, at rest)	85.3(77.9–94.2)	82.0(74.3–90.2)	87.9(78.9–97.1)	**<0.01**
Comorbidities				
DM, *n* (%)	77(20.8)	41(34.2)	36(14.3)	**<0.01**
MCTD^c^, *n* (%)	68(18.3)	33(27.5)	35(13.9)	**<0.01**
COPD, *n* (%)	6(1.6)	3(2.5)	3(1.2)	0.39
Emphysema	82(22.1)	29(24.2)	53(21.1)	0.51

Data were presented as mean ± SD, median (IQR) or *n* (%). RA, rheumatoid arthritis; ILD, interstitial lung disease; BMI, body mass index; ESR, erythrocyte sedimentation rate; DAS28-ESR, Disease Activity Score in 28 joints-Erythrocyte Sedimentation Rate; CRP, C-reactive protein; LDH, lactic dehydrogenase; RF, rheumatoid factor; CCP, cyclic citrullinated peptide; NLR, neutrophils/lymphocytes ratio; MLR, monocytes/lymphocytes ratio; PLR, platelet/lymphocyte ratio; SII, neutrophils × latelets/lymphocytes; SIRI, neutrophils × monocytes/lymphocytes; AISI, neutrophils × platelets × monocytes/lymphocytes; FVC, forced vital capacity; DLCO, diffusing capacity of the lungs for carbon monoxide; PaO2, partial pressure of oxygen in the arterial blood; DM, diabetes mellitus; MCTD, mixed connective tissue disease; COPD, chronic obstructive pulmonary disease. ^a^RA-ILD represented RA-pILD and RA-ILD patients. ^b^Seronegative RA represented RA patients with both negative RF and anti-CCP. ^c^MCTD, representing systemic autoimmune diseases other than RA, included Sjögren’s syndrome (*n* = 56), systemic lupus erythematosus (*n* = 4), idiopathic inflammatory myopathy (*n* = 7), and systemic sclerosis (*n* = 4) in this study. The bold values provided in tables mean that *P*-value<0.05 or 0.01.

### Imaging patterns at baseline and during follow-up among RA-ILD

**Figure 2:**
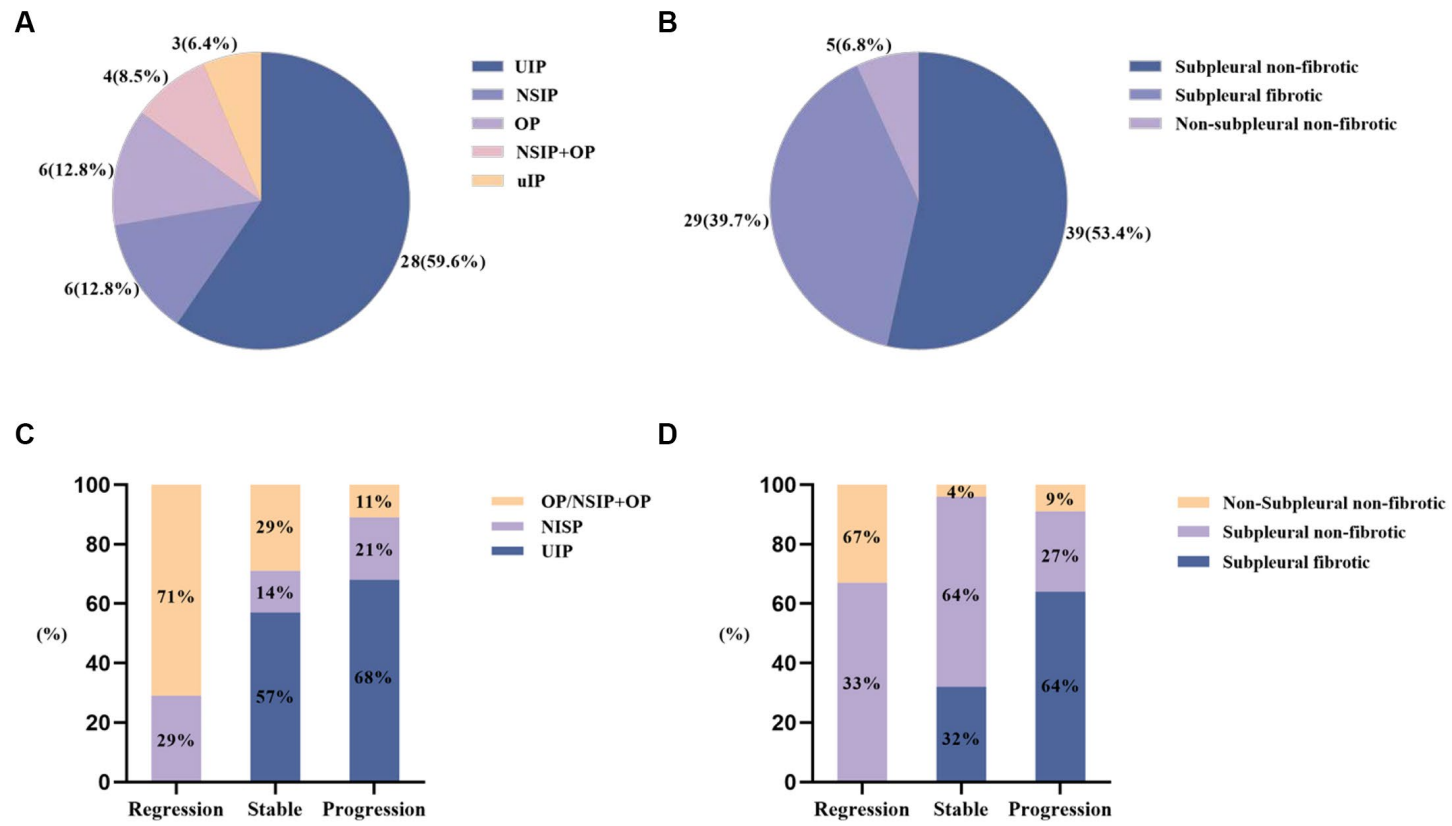
HRCT patterns at baseline and during follow-up among RA-ILD (including pILD and ILD). **(A)** HRCT patterns at baseline among RA-ILD; **(B)** HRCT patterns at baseline among RA-pILD; **(C)** HRCT patterns among RA-ILD during follow-up; **(D)** HRCT patterns among RA-pILD during follow-up.

As shown in Figure 2, the most prevalent imaging pattern observed in RA-ILD patients was usual interstitial pneumonia (UIP) (59.6%) among the 47 patients, while in RA-pILD patients, the most common pattern was subpleural non-fibrotic (53.4%) among the 73 patients. Among the progressors, 63.6% (14/22) exhibited subpleural fibrosis in RA-pILD and 67.9% (19/28) exhibited UIP in RA-ILD, which was the most frequent imaging patterns (see Figure 2 and Supplementary Table S2 for details).

### Follow-up of RA-ILD

Ninety-eight RA-ILD patients were followed up for a median duration of 19.1 (11.1–33.3) months, of whom 50 (50.1%) demonstrated fibrotic progression. As shown in Table 2, the progressors had a higher proportion of individuals aged >60.0 years, smokers, patients with DM, and those experiencing dyspnea and velcro crackles. Furthermore, the progressors exhibited higher HRCT scores at both baseline and follow-up compared to the non-progressors (all *p*< 0.05). Regarding the proportion of values above the upper limit of normal, the progressors showed higher levels of DAS28-ESR, ESR, CRP, LDH, and positive anti-CCP antibody (*p*< 0.01 or 0.05) compared to the non-progressors. Additionally, the progressors had higher levels of RF, NLR, SII, SIRI, and AISI compared to the non-progressors (*p*= 0.01 or *p*< 0.01).

**Table 2:** Baseline characteristics of RA-ILD stratified by progressors and non-progressors.

Variables	Pat. of follow-up	Progressor	Non-progressor	*P*-value
*n*	98	50	48	
Age, years	66.0(60.0–73.3)	67.0(62.0–75.3)	64.0(56.8–72.8)	0.07
Male, *n* (%)	34(34.7)	21(42.0)	13(27.1)	0.12
BMI, kg/m^2^	23.9 ± 3.5	23.2 ± 3.5	24.6 ± 3.5	0.06
Smoking status				**0.03**
Smoker, *n* (%)	33(33.7)	22(44.0)	11(22.9)	
Non-smoker, *n* (%)	65(66.3)	28(56.0)	37(77.1)	
Pack years	30.0(14.7–50.0)	40.0(18.8–52.5)	20.0(10.0–40.0)	0.19
Disease duration, ms	113.1(27.4–190.3)	96.8(15.7–191.6)	121.8(39.1–197.4)	0.39
Follow-up time, ms	19.1(11.1–33.3)	23.1(11.9–38.4)	15.5(9.7–31.8)	0.18
HRCT scores				
Baseline	6.5(4.0–8.3)	7.5(6.0–11.0)	5.0(3.0–7.8)	**<0.01**
Follow-up	7.0(3.0–11.0)	10.0(8.0–14.0)	4.0(2.0–6.0)	**<0.01**
Acute exacerbation	16(16.3)	11(22.0)	5(10.4)	0.12
Symptoms and signs, *n* (%)				
Dyspnea	40(40.8)	26(52.0)	14(29.2)	**0.02**
Cough	48(49.0)	28(56.0)	20(41.7)	0.31
Velcro crackles	37(37.8)	24(48.0)	13(27.1)	**0.03**
Laboratory findings				
DAS28-ESR	4.7 ± 1.9	5.7 ± 1.3	3.6 ± 1.7	**<0.01**
DAS28-ESR, *n* (%)				**<0.01**
≤5.1	51(52.0)	12(24.0)	39(81.3)	
>5.1	47(48.0)	38(76.0)	9(18.8)	
ESR, mm/h	24.0(12.5–42.3)	32.5(20.0–47.8)	20.0(7.0–35.8)	**<0.01**
ESR, *n* (%)				**<0.01**
≤15 mm/h	27(27.6)	5(10.0)	22(45.8)	
>15 mm/h	71(72.4)	45(90.0)	26(54.2)	
CRP, mg/dl	1.0(0.5–3.1)	2.4(0.9–5.3)	0.6(0.3–1.4)	**<0.01**
CRP, *n* (%)				**<0.01**
≤0.8 mg/dl	42(42.9)	12(24.0)	30(62.5)	
>0.8 mg/dl	56(57.1)	38(76.0)	18(37.5)	
Ferritin, *n* (%)				0.90
≤322 ng/ml	75(76.5)	38(76.0)	37(77.1)	
>322 ng/ml	23(23.5)	12(24.0)	11(22.9)	
LDH, U/L	216.0(178.8–275.0)	248.0(184.8–290.0)	207.0(177.3–234.0)	**0.02**
LDH, *n* (%)				**<0.01**
≤250 U/L	65(66.3)	25(50.0)	40(83.3)	
>250 U/L	33(33.7)	25(50.0)	8(16.7)	
RF positivity, *n* (%)	69(75.8)	40(80.0)	29(70.7)	0.30
RF titer, IU/ml	164.0(46.6–356.0)	227.5(93.3–441.0)	96.3(30.4–210.5)	**0.01**
Anti-CCP positivity, *n* (%)	78(79.6)	44(88.0)	34(70.8)	**0.04**
Anti-CCP titer, IU/ml	597.1(198.6–1278.6)	621.7(144.3–1702.0)	438.8(267.1–1136.7)	0.95
Seronegative RA^a^, *n* (%)	11(11.2)	4(8.0)	7(14.6)	0.30
NLR	3.3(2.2–4.1)	3.5(2.5–5.4)	2.7(2.0–3.5)	**<0.01**
MLR	0.3(0.2–0.4)	0.3(0.2–0.5)	0.3(0.2–0.4)	0.20
PLR	143.3(98.8–204.0)	150.7(98.1–254.9)	140.9(98.5–171.5)	0.22
SII	736.0(408.1–954.7)	805.8(551.8–1561.3)	533.4(367.2–880.8)	**<0.01**
SIRI	1.4(0.8–2.4)	1.5(1.0–3.2)	1.2(0.7–1.6)	**<0.01**
AISI	332.3(146.3–608.2)	390.9(172.8–793.5)	245.2(121.3–428.7)	**<0.01**
Pulmonary function				
FVC, %predicted	97.0 ± 20.5	91.4 ± 16.9	102.7 ± 22.3	**0.01**
DLCO, %predicted	66.1 ± 15.9	62.0 ± 14.2	70.1 ± 16.7	**0.02**
PaO2, mmHg (room air, at rest)	82.6(77.3–91.4)	81.0(76.2–88.1)	85.0(77.9–91.8)	0.08
Comorbidities				
DM, *n* (%)	34(34.7)	22(44.0)	12(25.0)	**0.048**
MCTD^b^, *n* (%)	27(27.6)	14(28.0)	13(27.1)	0.92
COPD, *n* (%)	2(2.0)	2(4.0)	0	0.50
Emphysema	24(24.5)	17(34.0)	7(14.6)	**0.03**

Data were presented as mean ± SD, median (IQR) or *n* (%). BMI, body mass index; ESR, erythrocyte sedimentation rate; DAS28-ESR, Disease Activity Score in 28 joints-Erythrocyte Sedimentation Rate; CRP, C-reactive protein; LDH, lactic dehydrogenase; RF, rheumatoid factor; CCP, cyclic citrullinated peptide; NLR, neutrophils/lymphocytes ratio; MLR, monocytes/lymphocytes ratio; PLR, platelet/lymphocyte ratio; SII, neutrophils × latelets/lymphocytes; SIRI, neutrophils × monocytes/lymphocytes; AISI, neutrophils × platelets × monocytes/lymphocytes; FVC, forced vital capacity; DLCO, diffusing capacity of the lungs for carbon monoxide; PaO2, partial pressure of oxygen in the arterial blood; DM, diabetes mellitus; MCTD, mixed connective tissue disease. ^a^Seronegative RA represented RA patients with both negative RF and anti-CCP. ^b^MCTD, representing systemic autoimmune diseases other than RA, included Sjögren’s syndrome (*n* = 56), systemic lupus erythematosus (*n* = 4), idiopathic inflammatory myopathy (*n* = 7), and systemic sclerosis (*n* = 4) in this study. The bold values provided in tables mean that *P*-value<0.05 or 0.01.

### Therapeutic regimens

In this cohort, RA patients had received at least one of the following treatments: nonsteroidal anti-inflammatory drugs (NSAIDs), glucocorticoids, conventional synthetic disease-modifying anti-rheumatoid drugs (csDMARDs), biological DMARDs (bDMARDs), and targeted synthetic DMARDs (tsDMARDs). The frequency of glucocorticoid use was higher in RA-ILD patients, while NSAIDs and csDMARDs were more frequently used in patients with RA alone (refer to Supplementary Table S3 for details). Among the follow-ups, there was a significant difference in the frequency of csDMARDs use between the progressors and non-progressors (see Supplementary Table S4 for more information).

### Risk factors for the prevalence and progression of RA-ILD

The statistically significant variables between the patients with RA-ILD or RA alone were considered for further analysis. Multiple logistic regression analysis identified age > 60.0 years (odds ratio [OR] 2.22, *p*< 0.01), smoking (OR 2.09, *p*= 0.045), DM (OR 3.09, *p*< 0.01), MCTD (OR 2.98, *p*< 0.01), LDH >250.0 U/L (OR 6.73, *p*< 0.01), and positive anti-CCP antibody (OR 2.06, *p*= 0.03) as independent risk factors for RA-ILD (Table 3).

**Table 3:** Logistic regression analysis for risk factors of the prevalence of RA-ILD.

	Univariate analysis		Multivariate analysis	
	OR(95%CI)	*P*-value	OR(95%CI)	*P*-value
Age > 60.0, years	2.76(1.73–4.41)	**<0.01**	2.22(1.29–3.80)	**<0.01**
Male	1.67(1.06–2.65)	**0.03**	1.04(0.48–2.23)	0.93
Smoking	2.02(1.25–3.24)	**<0.01**	2.09(0.94–4.62)	**0.045**
BMI, kg/m^2^	1.01(0.96–1.07)	0.62		
DM	2.56(1.51–4.33)	**<0.01**	3.09(1.70–5.64)	**<0.01**
MCTD	2.34(1.37–4.00)	**<0.01**	2.98(1.57–5.67)	**<0.01**
DAS28-ESR > 5.1	1.59(1.02–2.47)	**0.04**	1.05(0.62–1.78)	0.85
CRP > 0.8, mg/dl	1.03(0.66–1.61)	0.90		
ESR > 15 mm/h	1.03(0.62–1.70)	0.92		
Ferritin>322, ng/ml	2.11(1.21–3.67)	**<0.01**	1.54(0.80–2.96)	0.19
LDH > 250, U/L	7.85(4.05–15.21)	**<0.01**	6.73(3.26–13.91)	**<0.01**
RF	1.19(0.74–1.94)	0.47		
Anti-CCP antibody	2.14(1.22–3.75)	**<0.01**	2.062(1.090–3.902)	**0.03**
Hypoxemia^a^	1.70(1.07-2.69)	**0.02**	1.69(0.98–2.93)	0.06

BMI, body mass index; DM, diabetes mellitus; MCTD, mixed connective tissue disease; DAS28-ESR, disease activity score in 28 joints-Erythrocyte Sedimentation Rate; CRP, C-reactive protein; ESR, erythrocyte sedimentation rate; LDH, lactic dehydrogenase; RF, rheumatoid factor; CCP, cyclic citrullinated peptide. ^a^Hypoxemia representing PaO2 < 80 mmHg (room air, at rest). The bold values provided in tables mean that *P*-value<0.05 or 0.01.

**Figure 3:**
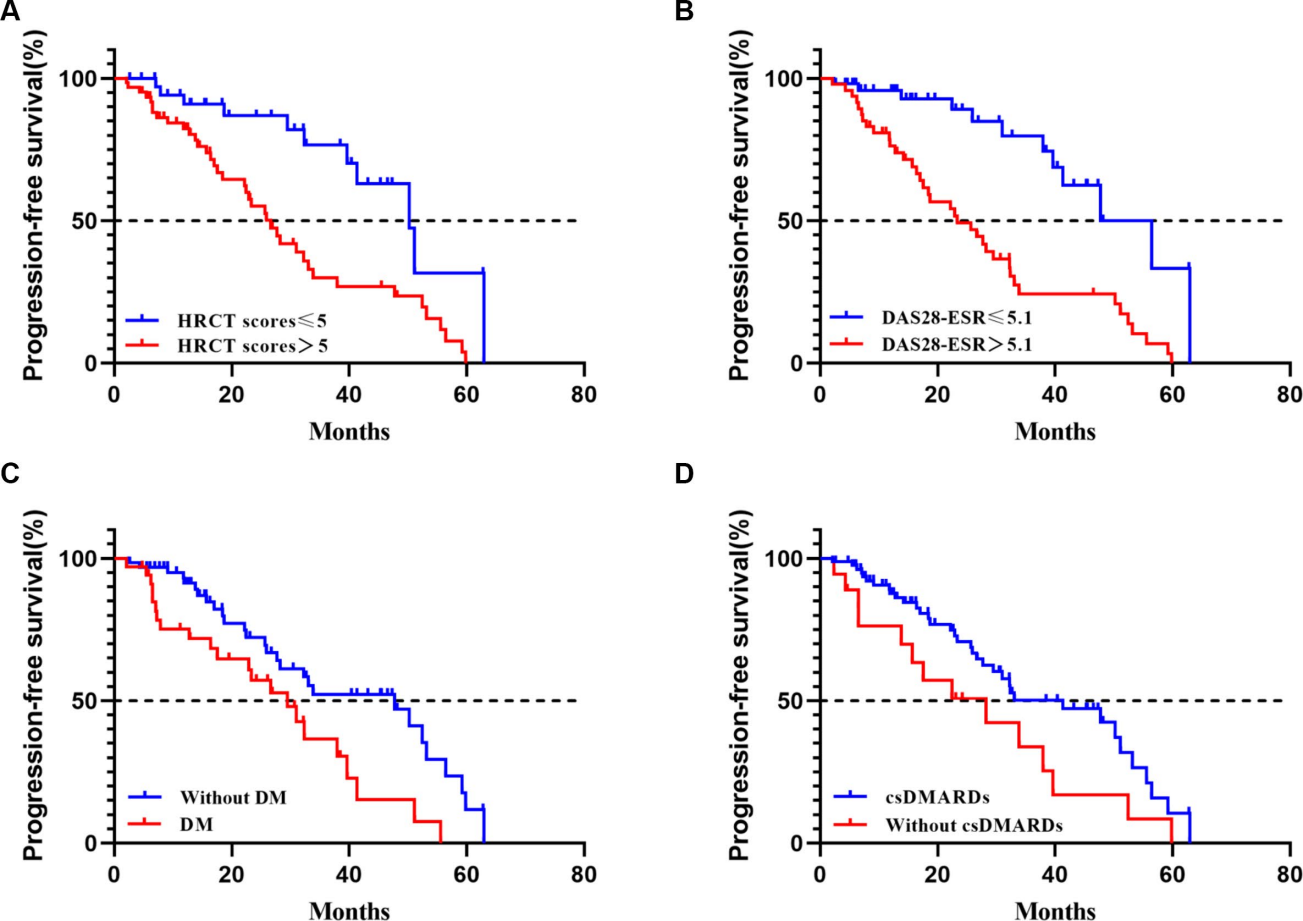
Progression-free survival in RA-ILD (including pILD and ILD). **(A)** progression-free survival according to HRCT scores at baseline with a 5 threshold (Log-rank test, *p*< 0.01); **(B)** progression-free survival according to DAS28-ESR at baseline with a 5.1 threshold (Log-rank test, *p*< 0.01); **(C)** progression-free survival according to with or without DM at baseline (Log-rank test, *p*= 0.01); **(D)** progression-free survival according to with or without use of csDMARDs at baseline (Log-rank test, *p*= 0.05).

The median progression-free survival [PFS] time was shorter in patients with DM (PFS 29.5 months), DAS28-ESR > 5.1 (PFS 23.3 months), and HRCT scores>5 (PFS 26.9 months) (see Figure 3). Subgroup analysis based on csDMARDs using the log-rank test showed no significant intergroup difference (*p*= 0.05) (refer to Figure 3 for visualization). Multiple Cox regression analysis identified DM (hazard ratio [HR] 2.47, 95% confidence interval [CI] 1.35–4.55, *p*< 0.01), DAS28-ESR > 5.1 (HR 2.32, 95% CI 1.02–5.26, *p*= 0.04), and HRCT scores>5 at baseline (HR 3.04, 95% CI 1.45–6.36, *p*< 0.01) as independent risk predictors for imaging progression of fibrosis in RA-ILD (including pILD and ILD) (Table 4). The complete information about which RA disease or ILD-related variables taken into account for regression analysis were also provided (Supplementary Tables S5, S6).

**Table 4:** Cox regression analysis for risk factors of the progression of RA-ILD.

	Univariate analysis		Multivariate analysis	
	OR(95%CI)	*P*-value	OR(95%CI)	*P*-value
Age > 60, years	1.46(0.71–3.03)	0.31		
Male	1.39(0.78–2.46)	0.26		
Smoking	1.38(0.77–2.46)	0.28		
BMI, kg/m^2^	1.20(0.42–3.44)	0.73		
DM	2.18(1.21–3.93)	**0.01**	2.47(1.35–4.55)	**<0.01**
MCTD	1.39(0.73–2.63)	0.31		
DAS28-ESR > 5.1	3.76(1.92–7.39)	**<0.01**	2.32(1.02–5.26)	**0.04**
CRP > 0.8, mg/dl	2.09(1.08–4.02)	**0.03**	1.03(0.49–2.18)	0.94
ESR > 15 mm/h	2.68(1.05–6.83)	**0.04**	1.64(0.57–4.70)	0.36
Ferritin>322, ng/ml	1.37(0.70–2.68)	0.37		
LDH > 250, U/L	1.44(0.81–2.56)	0.21		
RF	1.43(0.71–2.89)	0.32		
Anti-CCP antibody	1.50(0.59–3.84)	0.39		
Hypoxemia^a^	1.78(0.99-3.18)	0.05		
Baseline HRCT scores>5	3.15(1.57–6.36)	**<0.01**	3.04(1.45–6.36)	**<0.01**

BMI, body mass index; DM, diabetes mellitus; MCTD, mixed connective tissue disease; DAS28-ESR, disease activity score in 28 joints-Erythrocyte Sedimentation Rate; CRP, C-reactive protein; ESR, erythrocyte sedimentation rate; LDH, lactic dehydrogenase; RF, rheumatoid factor; CCP, cyclic citrullinated peptide. ^a^Hypoxemia representing PaO2 < 80 mmHg (room air, at rest). The bold values provided in tables mean that *P*-value<0.05 or 0.01.

## Discussion

This study provides a retrospective cohort analysis of patients with RA. The prevalence of ILD in RA patients varies widely, ranging from 1.0 to 58.0% in RA-ILD and 19.0 to 57.0% in RA-pILD patients (6). Previous studies reported abnormal chest HRCT findings in 15.0% of RA patients in the United States and interstitial lung changes in 22.0% of RA patients in Brazil (10, 20). In this cohort, 32.3% of RA patients had ILD (including pILD and ILD), with 19.7% classified as pILD and 12.7% as ILD. The difference in prevalence may be attributable to factors such as inpatient selection, older age, or different classification criteria for imaging description. Older male RA-ILD patients (>60.0 years) were more prevalent, consistent with previous findings (21, 22). While some patients had ILD prior to the RA diagnosis, many developed ILD as a secondary manifestation of RA. Nearly half of the RA-ILD patients, including pILD and ILD, did not exhibit respiratory symptoms or signs, with RA-pILD accounting for 83.3% of these patients. Therefore, it is recommended to perform routine chest HRCT scans in high-risk individuals (with connective tissue disease or familial ILD) to assess the presence or absence of ILD, irrespective of respiratory symptoms. Multidisciplinary discussions involving pulmonologists, rheumatologists, radiologists, and pathologists should be conducted if necessary for a comprehensive assessment (14).

The most common imaging pattern observed in RA-ILD patients was UIP, followed by nonspecific interstitial pneumonia (NSIP) (23, 24). However, the classification methods for pILD and ILD varied among studies, making it difficult to determine the exact proportion of each imaging pattern. HRCT currently remains the primary tool for identifying ILD in the absence of alternative biomarkers.

In this cohort, 51.0% of RA-ILD patients (including pILD and ILD) showed disease progression during a median follow-up period of 23.0 months. Among the RA-pILD patients, 23.0% were subsequently diagnosed with ILD, suggesting the importance of screening individuals with RA-pILD for RA-ILD in the future. Notably, RA-pILD patients who exhibited imaging regression (7 patients) showed improvement, with some transitioning to RA alone. Presence of DM, DAS28-ESR > 5.1, and baseline HRCT scores>5.0 were associated with imaging progression in RA-related pILD and ILD. Previous studies have also indicated that male gender, higher DAS28-ESR levels, HRCT-documented UIP-like fibrotic patterns, and higher baseline HRCT scores were risk factors for poor prognosis in RA-ILD (25, 26). Disease activity, as measured by DAS28-ESR, was strongly correlated with prognosis, with higher levels associated with increased risk of death (27). Identifying risk factors for imaging progression in RA related pILD and ILD could help identify patients with poor prognosis.

RA-ILD patients, including pILD and ILD, exhibited lower FVC% predicted and DLCO% predicted values compared to RA patients without ILD (26, 28–30). These findings suggest the presence of restrictive ventilatory dysfunction and decreased gas exchange capacity. Decline in FVC% predicted and DLCO% predicted values at baseline were independent predictors of worse survival in RA-ILD patients (30). Lower DLCO% predicted was particularly associated with increased risk of death (30). In this study, RA-ILD patients with progressive fibrotic CT scans showed lower FVC% predicted and DLCO% predicted values during follow-up.

The role of methotrexate in RA-ILD patients remains controversial. Some studies have shown that methotrexate is protective against the development of RA-ILD and delays its onset, while others have suggested that it increases the risk of ILD prevalence and progression (31, 32). Glucocorticoids are the mainstay treatment for RA-ILD, usually initiated with oral prednisone and tapered based on clinical response (6). However, glucocorticoids have been associated with increased risk of ILD prevalence and progression in RA patients (7). The choice of therapeutic drugs in this study was determined independently by the treating physicians based on the patients’ conditions.

This study provides valuable insights into the prevalence and clinical characteristics of pILD and ILD in patients with RA. It sheds light on the changes observed in RA related pILD and ILD patients and identifies potential risk factors for fibrosis progression in RA-ILD. However, it is important for readers to be mindful of the study’s limitations. Being a single-center study, there is a possibility of selection bias as only hospitalized individuals were included, which could have led to an overestimate of the prevalence of RA-ILD. Additionally, the retrospective design introduced some missing data and limited follow-up for certain patients, potentially introducing selection bias. The median follow-up duration of 19.1 months was relatively short for comprehensive evaluation of survival rates, overall mortality, ILD related mortality, and associated risk factors in RA-ILD patients. To obtain more precise and reliable results, prospective registration studies with larger sample sizes are necessary.

In summary, this study provides valuable insights into a hospital population-based cohort of RA patients, highlighting a significant prevalence of 32.3% for RA-ILD, encompassing both pILD and ILD. The presence and progression of ILD in RA patients are closely associated with a higher proportion of respiratory symptoms and signs, as well as impaired pulmonary function. Notably, almost half of the RA-ILD patients demonstrated imaging progression during the follow-up period. Risk factors for fibrosis progression in RA-ILD include DM, elevated DAS28-ESR levels, and advanced HRCT scores. Further investigations are warranted to better elucidate the roles of methotrexate and glucocorticoids in the management of RA-ILD.

## Data availability statement

The raw data supporting the conclusions of this article will be made available by the authors, without undue reservation.

## Ethics statement

The studies involving humans were approved by Ethics Committee of Beijing Chao-Yang Hospital. The studies were conducted in accordance with the local legislation and institutional requirements. Written informed consent for participation was not required from the participants or the participants’ legal guardians/next of kin in accordance with the national legislation and institutional requirements.

## Author contributions

DC: Conceptualization, Data curation, Formal analysis, Investigation, Methodology, Project administration, Resources, Software, Validation, Visualization, Writing – original draft. DS: Data curation, Formal analysis, Methodology, Writing – review & editing. YW: Data curation, Writing – review & editing. YS: Data curation, Writing – review & editing. NW: Writing – review & editing. QY: Conceptualization, Data curation, Formal analysis, Methodology, Project administration, Resources, Supervision, Validation, Visualization, Writing – review & editing.

## Abbreviations

RA: rheumatoid arthritis
ILD: interstitial lung disease
RF: rheumatoid factor
CCP: cyclic citrullinated peptide
IIP: idiopathic interstitial pneumonia
pILD: preclinical interstitial lung disease
HRCT: high resolution computed tomography
ILA: interstitial lung abnormality
IPF: idiopathic pulmonary fibrosis
PPF: progressive pulmonary fibrosis
DAS28-ESR: Disease Activity Score in 28 joints-Erythrocyte Sedimentation Rate
NLR: neutrophils/lymphocytes ratio
MLR: monocytes/lymphocytes ratio
PLR: platelet/lymphocyte ratio
SII: neutrophils×latelets/lymphocytes
SIRI: neutrophils×monocytes/lymphocytes
AISI: neutrophils×platelets×monocytes/lymphocytes
CRP: C-reactive protein
ESR: erythrocyte sedimentation rate
SD: standard deviation
IQR: interquartile range
DM: diabetes mellitus
LDH: lactate dehydrogenase
FVC: forced vital capacity
DLCO: diffusion capacity of the lungs for carbon monoxide
PaO2: partial pressure of arterial oxygen
UIP: usual interstitial pneumonia
NSAIDs: nonsteroidal anti-inflammatory drugs
csDMARDs: conventional synthetic disease modifying anti-rheumatic drugs
bDMARDs: biological disease modifying anti-rheumatic drugs
tsDMARDs: targeted synthetic disease modifying anti-rheumatic drugs
OR: odds ratio
PFS: progression free survival
HR: hazard ratio
CI: confidence interval
NSIP: nonspecific interstitial pneumonia
