# Supplementary material for: Progression of radiographic fibrosis in rheumatoid arthritis-associated interstitial lung disease
Source: Front Med (Lausanne). 2023 Sep 22;10:1265355. doi: 10.3389/fmed.2023.1265355 (PMC10556458; doi:10.3389/fmed.2023.1265355)
Supplement: Supplementary file 1 [file Presentation_1.pdf]

Progression of radiographic fibrosis in rheumatoid arthritis-associated interstitial lung disease

Dandan Chai[1], Di Sun[1], Yuanying Wang[1], Yawen Song[1], Na Wu[1,2], Qiao Ye[1,2]

[1] Clinical Center for Interstitial Lung Diseases, Beijing Institute of Respiratory Medicine, Beijing Chaoyang Hospital, Capital Medical University, Beijing, China

[2] Department of Occupational Medicine and Toxicology, Beijing Chaoyang Hospital, Capital Medical University, Beijing, China

Supplementary Materials

Contents

Appendix S1. Additional information of methods 3

Pulmonary function test

Chest high-resolution computed tomography (HRCT)

Description and scoring of chest HRCT

Laboratory findings

Appendix S2. Additional information of discussion 5

Risk factors for the prevalence and progression of rheumatoid arthritis-associated interstitial lung disease (RA-ILD)

References 6

Table S1 Demographics and baseline characteristics of RA-pILD and RA-ILD 8

Table S2 Chest HRCT changes during follow-up 10

Table S3 Therapeutic regimen of patients in RA-ILD 11

Table S4 Therapeutic regimen of progressors and nonprogressors in RA-ILD 12

Table S5 Logistic regression analysis for risk factors of the prevalence of RA-ILD 13

Table S6 Cox regression analysis for risk factors of the progression of RA-ILD 14

Figure S1 HRCT manifestations of a patient with progression of radiographic fibrosis in RA-pILD 15

Appendix S1. Additional information of methods

Pulmonary function test

Pulmonary function tests (PFTs) were conducted by certified technicians in accordance with the guidelines set by the American Thoracic Society (ATS) and European Respiratory Society (ERS). These tests included spirometry, whole-body plethysmography, and single-breath diffusing capacity for carbon monoxide measurements. The PFT data were analyzed and reported following the recommendations provided by the ATS/ERS guidelines.[1]

Chest high-resolution computed tomography (HRCT)

HRCT was performed using a GE Brightspeed 64-slice spiral CT scanner. The patient was positioned in a supine position with both hands raised over the head to maximize chest exposure and scapular spread. Continuous inhalation was performed during scanning, covering the lung apex to the costophrenic recess, followed by breath-holding. The scanning parameters were set as follows: voltage of 140 kV, current of 300 mA, scanning layer thickness of 5 mm, high-resolution layer thickness of 0.625 mm, spacing of 10 mm, bone algorithm reconstruction, and specific window settings for lung and mediastinal windows. The lung window had a window width of 1500 HU and a window position of 700 HU, while the mediastinal window had a window width of 400 HU and a window position of 40 HU. Two independent physicians interpreted the CT images, and any discrepancies were resolved through discussion to reach a consensus.

Description and scoring of chest HRCT

The chest HRCT images were described and scored by two blinded and experienced pulmonologists who were unaware of the clinical data. Interstitial lung disease (ILD) was categorized according to the idiopathic interstitial pneumonia (IIP) classification and 2018 IPF clinical practice guidelines, including usual interstitial pneumonia (UIP), nonspecific interstitial pneumonia (NSIP), organizing pneumonia (OP), NSIP+OP, and unclassified interstitial pneumonia (uIP).[2,3] Additionally, preclinical interstitial lung disease (pILD) was further divided into non-subpleural non-fibrotic, subpleural non-fibrotic, and subpleural fibrotic patterns based on the 2020 Fleischner Society Position Paper.[4] Each lung was divided into three zones by the levels of the inferior aortic arch and right inferior pulmonary vein. The degree of abnormal imaging was scored semiquantitatively based on the percentage of abnormal findings in each lung zone, using a scoring system ranging from 0 to 4 points for each zone, with a total score range of 0 to 24 points. The scoring method was consistent with previous studies, ensuring comparability.[5] The interobserver agreement, as measured by weighted kappa, was 0.83.

Laboratory findings

Laboratory tests included the detection of rheumatoid factor (RF) using an ELISA method with an RF IgM detection kit, and the testing of anti-citrullinated cyclic peptide (anti-CCP) antibodies using an ELISA method with an anti-CCP IgG detection kit.

Appendix S2. Additional information of discussion

Risk factors for the prevalence and progression of rheumatoid arthritis-associated interstitial lung disease (RA-ILD)

Previous studies have reported that male sex, age older than 50 years, smoking, longer disease duration, high Disease Activity Score in 28 joints-Erythrocyte Sedimentation Rate(DAS28) level, positive anti-CCP antibody and positive RF were risk factors for RA-ILD (including pILD and ILD).[6-10] Every 1 unit increase in DAS28 increased the risk of RA-ILD by 35.0%.[6] A prospective cohort study included 2328 patients with RA (median age 64 years, 89.3% male), 100 (4.3%) of whom had ILD at enrollment, seropositive (both RF and anti-CCP antibody positive) RA subjects had a higher risk of ILD than seronegative (both RF and anti-CCP antibody negative) subjects (OR 2.90, 95% CI 1.24-6.78, *P*=0.01), and anti-CCP antibody-positive (>15.0 U/mL) patients had an increased risk of ILD compared with anti-CCP antibody-negative patients (OR 1.91, 95% CI 1.61-7.18, *P*=0.01).[11]As for risk predictors of progression in RA-ILD, male, high DAS28-ESR level, UIP pattern, and high baseline HRCT score were the potential risk factors for disease progression.[12,13] A retrospective study in China showed that high DAS28-ESR level (OR 1.883, 95% CI 1.065–3.329, *P*=0.03), UIP pattern (OR 3.625, 95% CI 1.134–11.589, *P*=0.03), and high fibrosis (honeycombing and reticular abnormalities) scores in imaging (OR 1.791, 95% CI 1.029–3.115, *P*=0.04) were independent risk factors for the progression of RA-ILD.[13]Above all, several statistically significant variables were found, including age, sex, smoking, body mass index, diabetes mellitus, mixed

connective tissue disease, DAS28-ESR, C-reactive protein, erythrocyte sedimentation rate, ferritin, lactate dehydrogenase, RF, anti-CCP antibody and hypoxemia, for evaluating the risk predictors of the prevalence and progression of RA-ILD.

References

1. Graham BL, Steenbruggen I, Miller MR, et al. Standardization of spirometry 2019 Update. An official American Thoracic Society and European Respiratory Society technical statement. Am J Respir Crit Care Med, 2019,200(8): e70-e88.
2. Raghu G, Remy-Jardin M, Myers J L, et al. Diagnosis of idiopathic pulmonary fibrosis. An official ATS/ERS/JRS/ALAT clinical practice guideline. Am J Respir Crit Care Med, 2018,198(5): e44-e68.
3. Travis W D, Costabel U, Hansell D M, et al. An official American Thoracic Society/European Respiratory Society statement: Update of the international multidisciplinary classification of the idiopathic interstitial pneumonias. Am J Respir Crit Care Med, 2013,188(6):733-748.
4. Hatabu H, Hunninghake GM, Richeldi L, et al. Interstitial lung abnormalities detected incidentally on CT: a Paper from the Fleischner Society. Lancet Respir Med, 2020,8(7):726-737.
5. Nurmi H M, Kettunen H P, Suoranta S K, et al. Several high-resolution computed tomography findings associate with survival and clinical features in rheumatoid arthritis-associated interstitial lung disease. Respir Med, 2018,134:24-30.
6. Sparks J A, He X, Huang J, et al. Rheumatoid arthritis disease activity predicting incident clinically apparent rheumatoid arthritis-associated interstitial lung disease: A prospective cohort study. Arthritis Rheumatol, 2019,71(9):1472-1482.

7. Kamiya H, Panlaqui O M. Systematic review and meta-analysis of the risk of rheumatoid arthritis-associated interstitial lung disease related to anti-cyclic citrullinated peptide (CCP) antibody. BMJ Open, 2021,11(3): e40465.

8. Kakutani T, Hashimoto A, Tominaga A, et al. Related factors, increased mortality and causes of death in patients with rheumatoid arthritis-associated interstitial lung disease. Mod Rheumatol, 2020,30(3):458-464.

9. Assayag D, Lubin M, Lee J S, et al. Predictors of mortality in rheumatoid arthritis-related interstitial lung disease. Respirology, 2014,19(4):493-500.

10. Restrepo J F, Del R I, Battafarano D F, et al. Clinical and laboratory factors associated with interstitial lung disease in rheumatoid arthritis. Clin Rheumatol, 2015.0,34(9):15.029-15.036.

11. Natalini, J. G, Baker, J. F, Singh, N, et al. Autoantibody seropositivity and risk for interstitial lung disease in a prospective male-predominant rheumatoid arthritis cohort of U.S. veterans. Ann Am Thorac Soc, 18(4), 598-605.

12. Chen N, Diao C Y, Gao J, et al. Risk factors for the progression of rheumatoid arthritis-related interstitial lung disease: Clinical features, biomarkers, and treatment options. Semin Arthritis Rheum, 2022,55:15.02004.

13. Liu L, Fang C, Sun B, et al. Predictors of progression in rheumatoid arthritis-associated interstitial lung disease: A single-center retrospective study from China. Int J Rheum Dis, 2022,25(7):795-802.

Table S1 Demographics and baseline characteristics of RA-pILD and RA-ILD

Variables	All	RA-pILD	RA-ILD	*P*-value
n	120	73	47	
Age, yrs	66.7±11.1	66.3±10.8	67.3±11.5	0.63
Male, n (%)	46(38.3)	25(34.2)	21(44.7)	0.25
BMI, kg/m^2	24.2±3.6	24.5±3.6	23.7±3.6	0.26
Smoking status				0.77
Smoker, n (%)	44(36.7)	26(35.6)	18(38.3)	
Non-smoker, n (%)	76(63.3)	47(64.4)	29(61.7)	
Pack years	30.0(15.0-43.8)	24.0(14.8-32.5)	40.0(18.5-65.0)	0.04
HRCT scores	6.5(4.0-9.0)	4.0(3.0-7.5)	9.0(7.0-12.0)	**<0.01**
Symptoms and signs, n (%)				
Dyspnea	45(37.5)	11(15.1)	34(72.3)	**<0.01**
Cough	55(45.8)	24(32.9)	31(66.0)	**<0.01**
Velcro crackles	45(37.5)	15(20.5)	30(63.8)	**<0.01**
Laboratory findings				
DAS28-ESR	5.1(3.5-6.0)	5.0(3.5-6.1)	5.3(3.7-5.8)	0.98
DAS28-ESR, n (%)				0.44
≤5.1	64(53.3)	41(56.2)	23(48.9)	
>5.1	56(46.7)	32(43.8)	24(51.1)	
ESR, mm/h	26.0(16.0-44.0)	27.0(19.0-50.5)	23.0(10.0-39.0)	0.18
ESR, n (%)				0.11
≤15 mm/h	29(24.2)	14(19.2)	15(31.9)	
>15 mm/h	91(75.8)	59(80.8)	32(68.1)	
CRP, mg/dl	1.1(0.5-3.5)	2.0(0.6-5.2)	0.9(0.4-2.5)	**0.04**
CRP, n (%)				0.08
≤0.8mg/dl	47(39.2)	24(32.9)	23(48.9)	
>0.8mg/dl	73(60.8)	49(67.1)	24(51.1)	
Ferritin, ng/ml	180.0(110.5-332.2)	181.5(110.9-328.1)	178.6(109.1-336.4)	0.88
Ferritin, n (%)				0.91
≤322 ng/ml	90(75.0)	55(75.3)	35(74.5)	
>322 ng/ml	30(25.0)	18(24.7)	12(25.5)	
LDH, U/L	214.5(178.3-267.0)	208.0(176.5-260.5)	227.0(181.0-290.0)	0.18
LDH, n (%)				0.10
≤250 U/L	82(68.3)	54(74.0)	28(59.6)	
>250 U/L	38(31.7)	19(26.0)	19(40.4)	
RF positivity, n (%)	88(73.3)	53(72.6)	35(74.5)	0.82
RF titer, IU/ml	174.0(55.5-405.8)	174.0(55.5-487.5)	195.0(41.7-363.0)	0.69
Anti-CCP positivity, n (%)	101(84.2)	64(87.7)	37(78.7)	0.19
Anti-CCP titer, IU/ml	772.3(276.3-2294.5)	865.6(293.5-2753.5)	731.8(244.7-1700.0)	0.47

Seronegative RA [a], n (%)	11(9.2)	6(8.2)	5(10.6)	0.65
NLR	3.1(2.1-4.1)	3.0(2.0-4.1)	3.1(2.3-4.5)	0.64
MLR	0.3(0.2-0.4)	0.3(0.2-0.4)	0.3(0.2-0.4)	0.81
PLR	141.4(99.0-190.5)	143.8(105.0-211.9)	132.0(94.9-171.0)	0.35
SII	753.9(422.2-990.6)	765.0(378.8-1031.8)	719.8(452.9-951.6)	0.94
SIRI	1.4(0.8-2.4)	1.4(0.8-2.2)	1.4(0.8-2.9)	0.49
AISI	346.7(148.4-635.2)	346.7(122.4-547.6)	346.7(163.0-655.7)	0.57
Pulmonary function				
FVC, %predicted	96.3±20.8	103.7±20.1	87.6±18.2	**<0.01**
DLCO, %predicted	65.8±15.6	70.7±13.6	59.8±15.8	**<0.01**
PaO2, mmHg (room air, at rest)	82.0(74.3-90.2)	84.3(77.0-91.5)	80.1(71.2-85.2)	**0.04**
Comorbidities				
DM, n (%)	41(34.2)	24(32.9)	17(36.2)	0.71
Metformin, n (%)	5(4.2)	5(6.8)	0	0.16
MCTD [b], n (%)	33(27.5)	23(31.5)	10(21.3)	0.22
COPD, n (%)	3(2.5)	1(1.4)	2(4.3)	0.56
Emphysema	29(24.2)	18(24.7)	11(23.4)	0.88

Data were presented as mean±SD, median (IQR) or n (%).

Abbreviation: RA=rheumatoid arthritis; ILD=interstitial lung disease; BMI=body mass index; ESR=erythrocyte sedimentation rate; DAS28-ESR=Disease Activity Score in 28 joints-Erythrocyte Sedimentation Rate; CRP=C-reactive protein; LDH=lactic dehydrogenase; RF=rheumatoid factor; CCP=cyclic citrullinated peptide; NLR= neutrophils/lymphocytes ratio; MLR= monocytes/lymphocytes ratio; PLR= platelet/lymphocyte ratio; SII=neutrophils×latelets/lymphocytes; SIRI=neutrophils×monocytes/lymphocytes; AISI= neutrophils×platelets×monocytes/lymphocytes; FVC=forced vital capacity; DLCO=diffusing capacity of the lungs for carbon monoxide; PaO2= partial pressure of oxygen in the arterial blood; DM=diabetes mellitus; MCTD=mixed connective tissue disease.

[a] Seronegative RA represented RA patients with both negative RF and anti-CCP.

[b] MCTD, representing systemic autoimmune diseases other than RA, included Sjögren 's syndrome (n=22), systemic lupus erythematosus (n=4), idiopathic inflammatory myopathy (n=7), and systemic sclerosis (n=2) in this study.

Table S2 Chest HRCT changes during follow-up

Variables	All	RA-pILD	RA-ILD
n	98	56	42
Progression, n (%)	50(51.0)	22(39.3)	28(66.7)
Regression, n (%)	13(13.3)	6(10.7)	7(16.7)
Stable, n (%)	35(35.7)	28(50.0)	7(16.7)

Data were presented as n (%).

Abbreviation: RA=rheumatoid arthritis; ILD=interstitial lung disease; pILD=preclinical interstitial lung disease.

Table S3 Therapeutic regimen of patients in RA-ILD

Variables	All	RA-ILD [a]	RA alone	*P*-value
n	371	120	251	
NSAIDS, n (%)	176(47.4)	37(30.8)	139(55.4)	**<0.01**
Glucocorticoids, n (%)	135(36.4)	69(57.5)	66(26.3)	**<0.01**
csDMARDs, n (%)	349(94.1)	101(84.2)	248(98.8)	**<0.01**
Methotrexate, n (%)	232(62.5)	49(40.8)	183(72.9)	**<0.01**
Leflunomide, n (%)	82(22.1)	21(17.5)	61(24.3)	**<0.01**
Cyclophosphamide, n (%)	39(10.5)	36(30.0)	3(1.2)	**<0.01**
Hydroxychloroquine, n (%)	213(57.4)	46(38.3)	167(66.5)	**<0.01**
Sulfasalazine, n (%)	14(3.8)	4(3.3)	10(4.0)	1.00
bDMARDs [b], n (%)	13(3.5)	1(0.8)	12(4.8)	0.07
tsDMARDs [c], n (%)	5(1.3)	1(0.8)	4(1.6)	1.00

Data were presented as n (%).

[a] RA-ILD included RA-pILD and RA-ILD

[b] included adalimumab (n=6), golimumab (n=1) and tolimumab (n=1) in this study.

[c] included tofacitab (n=1) in this study.

Abbreviations: RA=rheumatoid arthritis; ILD=interstitial lung disease; pILD=preclinical interstitial lung disease; NSAIDS=nonsteroidal anti-inflammatory drugs; csDMARDs=conventional synthetic disease modifying anti-rheumatic drugs; bDMARDs=biological disease modifying anti-rheumatic drugs; tsDMARDs=targeted synthetic disease modifying anti-rheumatic drugs.

Table S4 Therapeutic regimen of progressors and nonprogressors in RA-ILD

Variables	Follow-up	Progressor	Nonprogressor	*P*-value
n	98	50	48	
NSAIDS, n (%)	27(27.6)	11(22.0)	16(33.3)	0.21
Glucocorticoids, n (%)	60(61.2)	35(70.0)	25(52.1)	0.07
csDMARDs, n (%)	80(81.6)	36(72.0)	44(91.7)	**0.01**
Methotrexate, n (%)	36(36.7)	19(38.0)	17(35.4)	0.79
Leflunomide, n (%)	16(16.3)	7(14.0)	9(18.8)	0.53
Cyclophosphamide, n (%)	32(32.7)	16(32.0)	16(33.3)	0.89
Hydroxychloroquine, n (%)	37(37.8)	15(30.0)	22(45.8)	0.11
Sulfasalazine, n (%)	3(3.1)	1(2.0)	2(4.2)	0.61
bDMARDs [a], n (%)	1(1.0)	0	1(2.1)	0.49
tsDMARDs [b], n (%)	1(1.0)	0	1(2.1)	0.49

Data were presented as n (%).

[a] included adalimumab (n=1) in this study

[b] included tofacitab (n=1) in this study

Abbreviations: RA=rheumatoid arthritis; ILD=interstitial lung disease; pILD=preclinical interstitial lung disease; NSAIDS=nonsteroidal anti-inflammatory drugs; csDMARDs=conventional synthetic disease modifying anti-rheumatic drugs; bDMARDs=biological disease modifying anti-rheumatic drugs; tsDMARDs=targeted synthetic disease modifying anti-rheumatic drugs.

Table S5 Logistic regression analysis for risk factors of the prevalence of RA-ILD

	Univariate analysis		Multivariate analysis	
	OR（95%CI）	*P*-Value	OR（95%CI）	*P*-Value
Age>60.0, yrs	2.76(1.73-4.41)	**<0.01**	1.94(1.09-3.45)	**0.03**
Male	1.67(1.06-2.65)	**0.03**	0.96(0.42-2.23)	0.93
Smoking	2.02(1.25-3.24)	**<0.01**	2.02(0.87-4.67)	0.10
BMI, kg/m^2	1.01(0.96-1.07)	0.62		
DM	2.56(1.51-4.33)	**<0.01**	3.16(1.67-6.01)	**<0.01**
MCTD	2.34(1.37-4.00)	**<0.01**	2.02(1.00-4.05)	**0.049**
DAS28-ESR>5.1	1.59(1.02-2.47)	**0.04**	1.30(0.74-2.30)	0.37
CRP>0.8, mg/dl	1.03(0.66-1.61)	0.90		
ESR>15, mm/h	1.03(0.62-1.70)	0.92		
Ferritin>322, ng/ml	2.11(1.21-3.67)	**<0.01**	1.65(0.81-3.36)	0.17
LDH>250, U/L	7.85(4.05-15.21)	**<0.01**	5.60(2.49-12.60)	**<0.01**
RF	1.19(0.74-1.94)	0.47		
Anti-CCP antibody	2.14(1.22-3.75)	**<0.01**	2.10(1.06-4.16)	**0.03**
NLR	1.14(1.02-1.26)	**0.02**	1.08(0.86-1.37)	0.50
MLR	2.54(0.54-12.05)	0.24		
PLR	1.00(1.00-1.00)	**0.049**	1.00(1.00-1.00)	0.05
SII	1.00(1.00-1.00)	0.42		
SIRI	1.33(1.11-1.60)	**<0.01**	1.08(0.80-1.45)	0.63
AISI	1.00(1.00-1.00)	0.12		
FVC, %predicted<70%	1.41(0.52-3.81)	0.50		
DLCO, %predicted<70%	1.59(0.67-4.21)	0.43		
Hypoxemia [a]	1.70(1.07-2.69)	**0.02**	1.42(0.80-2.54)	0.24
Glucocorticoids	2.27(1.44-3.55)	**<0.01**	1.40(0.81-2.40)	0.23
Methotrexate	0.24(0.15-0.38)	**<0.01**	0.28(0.16-0.49)	**<0.01**

Abbreviations: BMI=body mass index; DM=diabetes mellitus; MCTD=mixed connective tissue disease; DAS28-ESR=disease activity score in 28 joints-Erythrocyte Sedimentation Rate; CRP=C-reactive protein; ESR=erythrocyte sedimentation rate; LDH=lactic dehydrogenase; RF=rheumatoid factor; CCP=cyclic citrullinated peptide. NLR= neutrophils/lymphocytes ratio; MLR= monocytes/lymphocytes ratio; PLR= platelet/lymphocyte ratio; SII=neutrophils×latelets/lymphocytes; SIRI=neutrophils×monocytes/lymphocytes; AISI= neutrophils×platelets×monocytes/lymphocytes; FVC=forced vital capacity; DLCO=diffusing capacity of the lungs for carbon monoxide.

[a] Hypoxemia representing PaO2<80mmHg (room air, at rest).

Table S6 Cox regression analysis for risk factors of the progression of RA-ILD

	Univariate analysis		Multivariate analysis	
	OR（95%CI）	*P*-Value	OR（95%CI）	*P*-Value
Age>60, yrs	1.46(0.71-3.03)	0.31		
Male	1.39(0.78-2.46)	0.26		
Smoking	1.38(0.77-2.46)	0.28		
BMI, kg/m^2	1.20(0.42-3.44)	0.73		
DM	2.18(1.21-3.93)	**0.01**	2.08(1.08-4.04)	**0.03**
MCTD	1.39(0.73-2.63)	0.31		
DAS28-ESR>5.1	3.76(1.92-7.39)	**<0.01**	1.74(0.74-4.13)	0.21
CRP>0.8, mg/dL	2.09(1.08-4.02)	**0.03**	1.31(0.58-2.96)	0.52
ESR>15, mm/h	2.68(1.05-6.83)	**0.04**	1.80(0.59-5.47)	0.30
Ferritin>322, ng/mL	1.37(0.70-2.68)	0.37		
LDH>250, U/L	1.44(0.81-2.56)	0.21		
RF	1.43(0.71-2.89)	0.32		
Anti-CCP antibody	1.50(0.59-3.84)	0.39		
NLR	1.06(1.01-1.12)	**0.03**	1.00(0.91-1.11)	0.93
MLR	5.52(1.00-30.73)	0.05		
PLR	1.00(1.00-1.00)	0.19		
SII	1.00(1.00-1.00)	0.06		
SIRI	1.29(1.11-1.49)	**<0.01**	1.28(0.87-1.86)	0.21
AISI	1.00(1.00-1.00)	**<0.01**	1.00(1.00-1.00)	0.66
FVC, %predicted<70%	2.17(0.85-5.56)	0.11		
DLCO, %predicted<70%	2.17(0.85-5.56)	0.11		
Hypoxemia[a]	1.70(1.07-2.69)	**0.02**	0.96(0.49-1.86)	0.90
Glucocorticoids	2.58(1.37-4.86)	**<0.01**	1.61(0.81-3.19)	0.18
Methotrexate	0.72(0.40-1.30)	0.28		
Baseline HRCT scores>5	3.15(1.57-6.36)	**<0.01**	2.69(1.20-6.03)	**0.02**

Abbreviations: BMI=body mass index; DM=diabetes mellitus; MCTD=mixed connective tissue disease; DAS28-ESR=disease activity score in 28 joints-Erythrocyte Sedimentation Rate; CRP=C-reactive protein; ESR=erythrocyte sedimentation rate; LDH=lactic dehydrogenase; RF=rheumatoid factor; CCP=cyclic citrullinated peptide. NLR= neutrophils/lymphocytes ratio; MLR= monocytes/lymphocytes ratio; PLR= platelet/lymphocyte ratio; SII=neutrophils×latelets/lymphocytes; SIRI=neutrophils×monocytes/lymphocytes; AISI= neutrophils×platelets×monocytes/lymphocytes; FVC=forced vital capacity; DLCO=diffusing capacity of the lungs for carbon monoxide.

[a] Hypoxemia representing PaO2<80mmHg (room air, at rest).

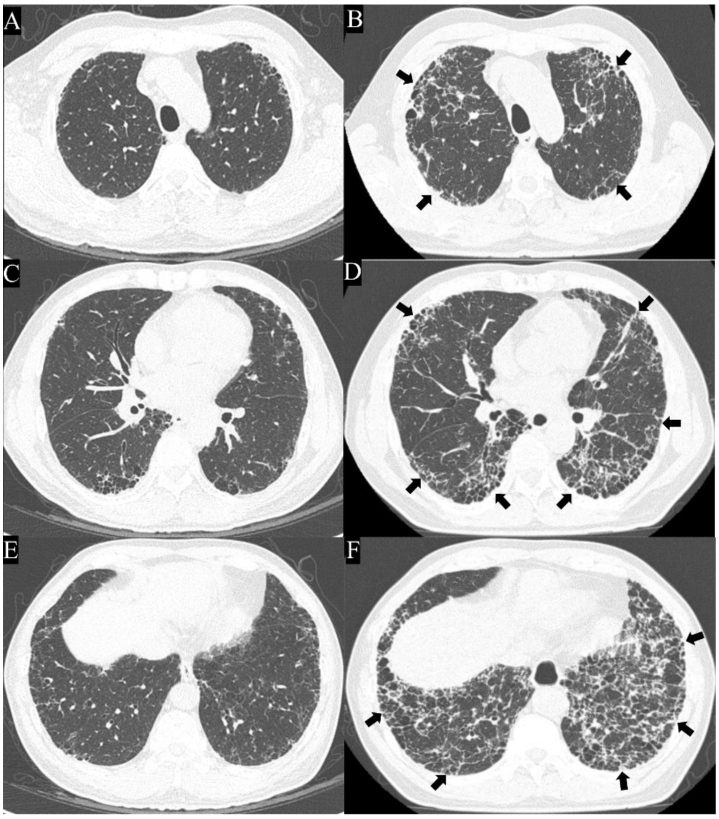

Figure S1. HRCT manifestations of a patient with progression of radiographic fibrosis in RA-pILD

A 73-year-old male with a 16-year history of rheumatoid arthritis (RA) was included in this study. Upon enrollment, the patient underwent chest high-resolution computed tomography (HRCT), revealing distinct imaging findings at three different levels (A, C, E). The HRCT images demonstrated prominent bronchovascular bundles and thickened interlobular septa in both lungs. Additionally, multiple fine reticulations were observed in the subpleural region, indicating a subpleural fibrotic pattern. Following a 30-month follow-up period, the patient underwent a repeat chest HRCT (B, D, F), which showed worsening abnormalities compared to the initial assessment. Specifically, the lower lung zone exhibited an imaging pattern consistent with usual interstitial pneumonia.
